# Therapeutic trajectories of families with rare diseases in Chile from the perspectives of patients, carers, and healthcare workers: a qualitative study

**DOI:** 10.1186/s13023-025-03595-6

**Published:** 2025-02-25

**Authors:** Báltica Cabieses, Alexandra Obach, Antonia Roberts, Gabriela Repetto

**Affiliations:** 1 Centro de Salud Global Intercultural, Instituto de Ciencias e Innovación en Medicina, Facultad de Medicina Clínica Alemana, Facultad de Psicología, Universidad del Desarrollo, Santiago, Chile; 2 Department of Health Sciences, University of York, York, UK; 3 Programa de Enfermedades Raras, Centro de Genómica y Genética, Instituto de Ciencias e Innovación en Medicina, Facultad de Medicina Clínica Alemana, Universidad del Desarrollo, Santiago, Chile

**Keywords:** Rare diseases, Therapeutic trajectories, Qualitative analysis, Chile, Perceptions and experiences, Healthcare systems

## Abstract

**Background:**

Rare diseases are conditions that have a low prevalence in the population and a high disease burden and are often chronic and progressive. International evidence concerning the experience of people and families living with rare diseases is scarce, leading to late and erroneous diagnoses, as well as non-specific treatments. This study explored the therapeutic trajectories of people and families living with rare diseases within Chile’s public and private healthcare systems from the perspective of patients, caregivers, and medical teams, including the initial symptoms, first consultation, testing, diagnosis, treatment, and follow-up.

**Methods:**

A qualitative exploratory study was conducted through multiple case studies. Sixty participants were interviewed in person and/or virtually: patients (*n* = 16), caregivers (*n* = 22), healthcare workers (*n* = 20), and two patient organisation leaders. The material was analysed using thematic analysis. The project was approved by the Scientific Ethics Committee of Facultad de Medicina Clínica Alemana, Universidad del Desarrollo.

**Results:**

After similar initial symptoms and first consultation, three main types of trajectories were identified: (i) the path taken by those who reach a diagnosis for a disease that has specific treatment available; (ii) the journey of those who reach a diagnosis for their health condition, but their disease does not have a specific treatment available; and (iii) the trajectory of those who have not reached a diagnosis and receive symptomatic treatments for symptoms.

**Conclusions:**

The therapeutic trajectories of patients with rare symptoms are similar in terms of initial symptoms and first consultation. However, their paths diverge at the diagnostic stage, with diverse experiences related to these journeys, largely based on having a diagnosis and whether there is a specific treatment. Rare conditions in Chile requires further attention and urgent action that considers those who live with them and their families.

**Supplementary Information:**

The online version contains supplementary material available at 10.1186/s13023-025-03595-6.

## Background

Rare Diseases (RD) pose challenges to patients, clinicians and the general population due to their low prevalence. Lack of awareness can result in delayed or incorrect diagnoses and treatments. RDs have strong economic and social impacts on the people who live with them and on health systems. The associated costs may be greater when there is no specific treatment and when the treatment is delayed, leading to health complications and deterioration. Similarly, people who suffer from RDs often face invisibility and discrimination in a variety of domains, such as health, education, and work. These impacts add to the costs associated with managing their disease, which can plunge them into a cycle of vulnerability and poverty [1].

In Chile, it is estimated that approximately one million people live with a rare disease [2]. Therefore, although the prevalence of each disease is low, cumulatively, they have a significant impact on population health [3]. Despite these estimates, Chile has no public policy or specific legislation broadly for addressing RDs. Some particular regulations and programs contain therapeutic guidelines for a small number of RDs [4, 5]. These programs grant some financial protection and pathways in the Chilean health system, which is a mix [6] comprising of the public sector (80%) that covers the sickest and poorest population and the private sector that covers those with the greatest resources and the youngest and/or healthiest population. These two systems entail great differences in timing, access to specialists, and out-of-pocket expenses, which impact the experiences of patients.

The concepts of trajectories, flows, navigation, or journeys are used to understand and characterise the complex paths patients must follow inside and outside the health system when experiencing an RD. Approaches to therapeutic trajectories are made from different disciplines with some distinct emphases. In the health domain, there is not one standardised definition for the concept of a trajectory. There are models that emphasise the key events that the patient experiences during the trajectory, such as the identification of body changes, the initial health consultations, and access to diagnosis and therapies [7, 8]. In contrast, other models focus on identifying delays in the phases where decisions are made during the trajectory [9–11].

Therapeutic trajectories study the paths that patients and their families follow in a series of diseases requiring constant interactions with the health system, such as cancer [15–17] and other diseases [18–23]. Despite the significance of this approach to health system performance and quality of care for many different health conditions, it has been poorly researched in Chile and Latin America, especially for RDs [14]. In the social sciences, a person’s process of identifying a health problem and its subjective meaning can respond to their particular contextual interpretation frameworks [12, 13]. From this perspective, routes that go beyond doctor-patient interactions are incorporated, and the search for solutions through traditional, complementary, and alternative healing strategies depending on individuals’ values and beliefs is considered [12].

By studying therapeutic trajectories, the experiences of patients and their families when interacting with the health system or other actors outside the system can be better understood. This type of knowledge sheds light on the unique paths that individuals follow to understand and manage their health conditions [14]. Incorporating the voices of patients, caregivers, and health teams to reconstruct these care routes allows us to identify critical nodes in the care and management of these complex RD cases that tend to be overlooked by other research methodologies [14]. Hence, investigating the subjective trajectories of people with RD in Chile may contribute to generating solutions anchored in people’s experiences and underserved needs and improve the health system’s management and quality of care. This study explored the therapeutic trajectories of RDs within Chile’s public and private healthcare systems by describing the experiences and perceptions of patients, caregivers, and medical teams with respect to several trajectory milestones, including the initial symptoms, first consultation, testing, diagnosis, treatment, and follow-up.

## Methods

### Study setting

This work focused on a qualitative exploration of the therapeutic trajectories of patients with an RD in Chile, according to several relevant actors: the patients themselves, their caregivers, and healthcare teams.

### Study design

A qualitative methodological approach was used to obtain an image of the object of study, approaching the natural context of the experience with diseases through the discourses of patients, caregivers, and health teams [24]. This exploratory study sought to investigate an experience that has not been widely scrutinised scientifically. It was conducted through a multiple case study, a qualitative methodological design in which the researcher explores multiple bounded systems (i.e., several cases) through in-depth data collection. Case studies involve various sources of information to provide a detailed description of the case [24], and their relevance lies in the fact that they allow a deep understanding of the topic of study [25].

### Participant recruitment and selection

Participant recruitment was based on theoretical and practical criteria, considering the feasibility of conducting interviews with the subjects that reflected the study objective. Access to study participants (patients, caregivers, and health teams) in Chile was achieved through various strategies between April 2022 and March 2023:

- Patients from an exome sequencing study of undiagnosed diseases [26] were invited to participate. A total of 138 participants were included in this exome study, seven of whom were adults who did not have any neurological or speech disabilities. Five patients agreed to participate. Additionally, links and dissemination flyers were distributed to patient organisations to recruit other patients with an RD. Participants from previous studies conducted by the authors were also contacted. Through this recruitment strategy, an additional 11 participants with RDs were interviewed. A total of 16 patients who were recruited from these strategies participated.
- For caregivers, companions of participants were referred by patients from the exome sequencing study. Eight caregivers agreed to participate. Additionally, links and dissemination flyers were distributed to patient organisations to recruit caregivers. Participants from other related projects led by the research team and/or contacted through patient organisations were also interviewed. A total of 14 caregivers agreed to participate.
- For healthcare teams, technicians and professionals referred by patients and caregivers as part of their health teams were interviewed. Seven professionals agreed to participate. A convenience approach was taken to contact people who work on RDs in Chile. Thirteen additional professionals agreed to participate.

In summary, a total of sixty participants were interviewed. Among them, 16 were patients, of whom 11 had a specific diagnosis and five were undiagnosed. There were 22 caregivers, of whom 11 had a family member with a diagnosis and 11 had a family member without a diagnosis. There were 20 healthcare workers, of whom 14 were doctors, three were nurses, and three were other types of healthcare professionals. Additionally, two patient association leaders, one from an umbrella patient organisation and one from a specific disease organisation, were interviewed.

The initial strategy considered the generation of triads between patients, caregivers, and healthcare professionals, with the purpose of highlighting the therapeutic trajectories of patients from the perspectives of these three social actors in each case. We were able to form seven such triads. In other cases, it was not possible for various reasons, including patients who had lost contact with their health teams or had experienced stress in their relationships with them. Therefore, nine dyads of patients and carers were achieved for the remaining cases, and the remaining participants were considered individually (Table 1).

### Data collection

Semi-structured interviews were conducted, which consisted of a conversation between the researcher and the reporting subject [27]. These interviews were conducted in person or virtually through the Zoom platform, depending on the interviewee’s comfort and geographical proximity to the research team. The interviews were conducted in Spanish and then translated into English. For the interviews with the various participants, interview scripts were constructed based on the main topics that guided the research, including the general experience with the disease, the therapeutic itineraries from the onset of symptoms to the present, and the diagnosis and treatment phase.

### Data analysis

The interviews were audio recorded and transcribed verbatim into a Microsoft Word document. Each transcript was checked for accuracy against the original recording. Each interviewee was assigned a code to protect the confidentiality of the participants. Three researchers (AO, BC, and AR) carried out separate inductive thematic analyses [27] of the interviews, identifying patterns and themes derived from the data. All the researchers familiarised themselves with the data by first reading the transcribed interviews. Then, they carried out an open coding process by identifying concepts connected by similarities or differences within the raw data. These concepts were then translated into codes and subcodes within overarching categories. The researchers then reviewed the main categories, codes, and subcodes, each of which was identified separately. They undertook a consensus-building process, discussed discrepancies, and agreed on a final version. The analysis was supported by the Atlas.ti 8 software. The verbatim quotations selected for this article were translated from Spanish by a native English speaker translator and checked by the authors to verify that the translations captured the original meaning. The selection of citations was made based on their relevance to the topics to be addressed in this article.

### Ethical considerations

The study was approved by the Ethics Committee of Facultad de Medicina, Clínica Alemana Universidad del Desarrollo. Participation was voluntary, and participants filled out an informed consent form available online or on paper before taking part in the interview, securing written informed consent. All the data were recorded anonymously, and no information allowing identification of the participants was kept except for the consent forms, which are stored on the PI’s computer in a locked file.

## Results

Seventy**-**five% of the participants were women, and 25% were men. Among patients and caregivers, 68% had public health coverage, and 32% had private health coverage. Among the health workers, 50% were employed in the public health system, 15% in the private health system, and 35% in both systems. The majority of the caregivers (82%) were mothers of a patient, and 25% of the health workers were treating a patient involved in the research. Table 1 shows the sociodemographic characteristics of the participants.

**Table 1:** Sociodemographic characteristics of the participants (*n* = 60)

Patients (*N* = 16)		
Characteristic		Frequency (n)
Gender	Female	10
	Male	6
Health Coverage	Public	10
	Private	6
Diagnosis	Yes	12
	No	4
Age range (years)	Adolescents	3
	Adults	13
**Caregivers (N = 22)**		
Characteristic		Frequency (n)
Gender	Female	19
	Male	3
Health Coverage	Public	16
	Private	6
Relationship with the patient with an RD	Mother	18
	Partner	2
	Other family member	2
**Healthcare workers (N = 20)**		
Characteristic		Frequency (n)
Gender	Female	15
	Male	5
Occupation	Geneticist	8
	Paediatrician	5
	Other areas	7
Health system	Public	10
	Private	3
	Both	7
Treating a patient involved in the research	Yes	5
	No	15
**Leaders of patient organisations (N = 2)**		
Characteristic		Frequency (n)
Gender	Female	1
	Male	1
Role regarding RD	Patient	1
	Caregiver	1
Type of organisation	Umbrella organisation	1
	Specific disease organisation	1
**Total**		*N* = 60

### Types of trajectories

**Fig. 1:**
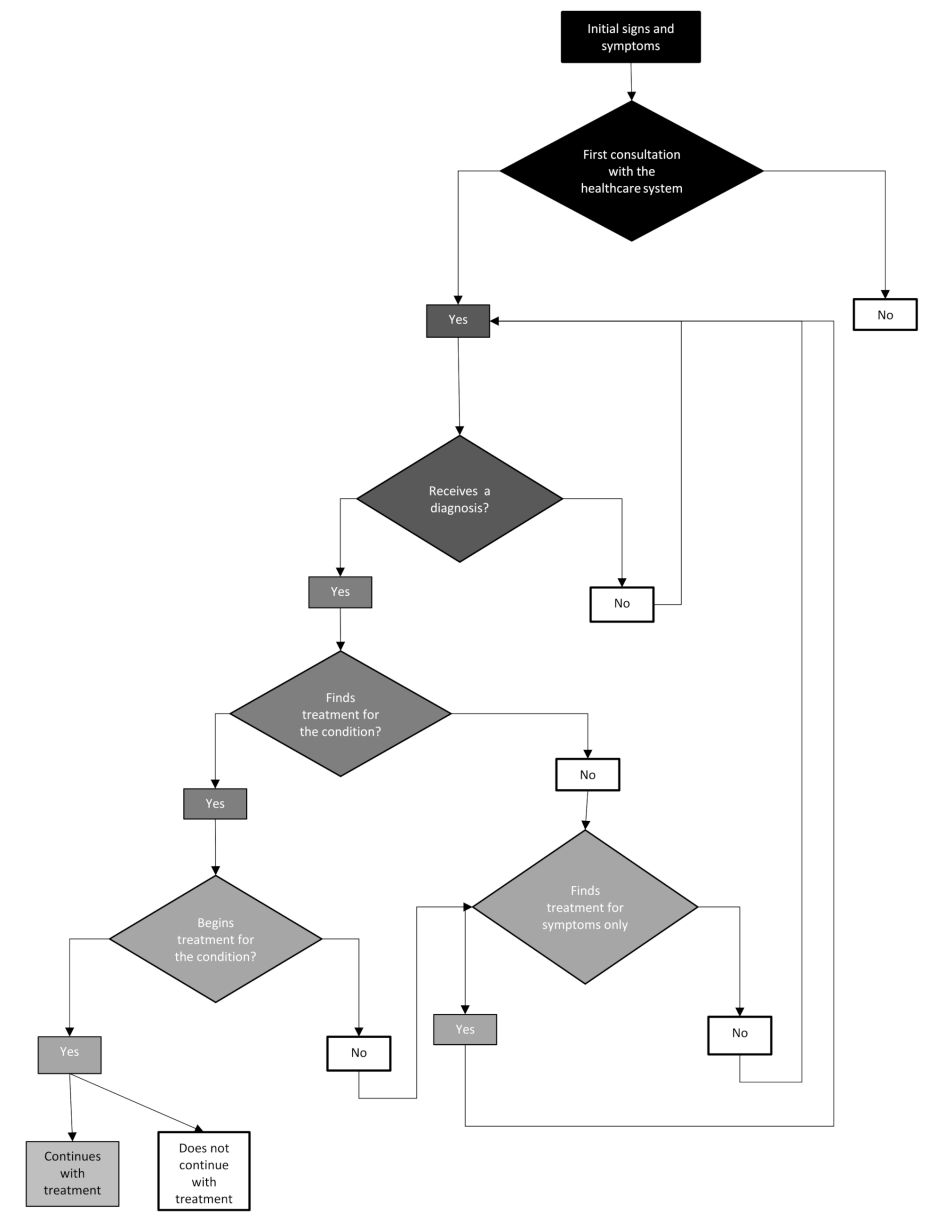
Therapeutic trajectory flowchart of patients with rare diseases in Chile. Legend: Fig. 1 summarises the therapeutic trajectories of people living with rare diseases in Chile’s health system. Each diamond symbolises significant milestones in the trajectory that imply decisions, such as initial consultation with the health system, reaching a diagnosis, reaching treatment for the condition or treatment for the symptoms. Each diamond symbolises significant milestones in the trajectory that imply decisions, such as initial consultation with the health system, reaching a diagnosis, reaching treatment for the condition or treatment for the symptoms. For each of these milestones, individuals can obtain a favourable outcome symbolised by a yes, which opens a process of moving forward on the trajectory, or an unfavourable one with a no. The arrows emerging from the cells labelled “no” illustrate the continuous re-consultation processes undertaken by patients and their families in the search for answers to their health conditions. The darker shades at the beginning of the figure symbolise the steps of the trajectory where there are more patients, subsequently decreasing to the following steps due to those who do not receive adequate responses to their condition

Despite the variability inherent in health conditions, three types of trajectories of patients with rare diseases were identified: (i) those who had a diagnosis for a disease that has specific treatment available for it, or treatment that manages to normalise the symptoms and reduce the person’s discomfort; (ii) those who reach a diagnosis for their health condition, but their disease does not have a specific treatment available, and the symptomatic treatments they receive fail to make the health condition more bearable; and (iii) those who did not reached a diagnosis and are still in the search process, where there may be trials of treatments for symptoms and different suspected diagnoses. Regardless of the type of condition, these three trajectories are similar at the initial symptoms, first consultation, and diagnosis seeking stages. However, their paths separate from the aetiological diagnostic result stage onward, with diverse experiences related to these journeys, mainly based on whether they had a diagnosis and were receiving treatment. Figure 1 displays the combined trajectories of all patients.

### First symptoms

The initial appearance of signs and symptoms varies, but the common characteristic is that the process of identifying symptoms is unique, depending on the person´s developmental stage. When symptoms appeared in childhood, it was evident that patients had no notion about the initial search process conducted by their caregivers, who were generally their parents, who noticed the symptoms and experienced the impact of the initial search processes:

“I was diagnosed when I was two years old, so I didn’t realise it; it was my parents and especially my mother […] that stage, more than anything, they lived through, I kind of didn’t realise anything, and the first symptoms were, of course, that I was not eating […]” (Patient with a diagnosis).

According to the interviewees, the beginning of their trajectories were marked by an increase in unspecific symptoms that gradually appeared from adolescence to adulthood, were often mild and similar to common health conditions, and thus went unnoticed in examinations and medical consultations:

“Before I got married, I wasn’t worried. Then, I started to worry because I saw that my skin was becoming more and more lax and elastic. It seemed that in adolescence, this was increasing, and in my youth, I was just married; I got married very young, so that was an issue because I began to realise that this was already signifying a deterioration” (Patient without a diagnosis).

In some cases, participants reported the appearance of sudden and severe signs and symptoms that appeared in adolescence or adulthood and escalated quickly and resulted in hospitalisation. In these cases, patients experienced an acute life transformation:

“I was driving, with the sweat, sitting for so many hours, those blisters were bursting, and when we got to Concepción, I remember that we arrived tired, we went to bed in the hotel, and when I got up the next day, much of my skin had remained on the sheet. The sheets were blood, blood everywhere. I couldn’t even wear underwear anymore, because also my groin, everything was completely torn, without skin” (Patient with a diagnosis).

In this initial stage of the trajectory, the participants noted the importance of support networks in investigating their unusual symptoms and motivating them to begin the process of consultation and search for answers to the health situation. These support networks can be made up of family, friends, work or educational networks, among others:

“One day, I was at home with my dad, and I yawn, right? My dad sees something strange in my mouth, calls my mother, and says, “China, you know what? The girl has something in her mouth.” They made me yawn again, of course, and there they saw my soft palate, right? I was born without the bell rather […]” (Caregiver of a patient without a diagnosis).

When relatives had similar symptoms, the symptoms were investigated faster; therefore, the trajectories began sooner. In these cases, access to information about the family history and the diseases to which the symptoms may correspond would be essential for the initial phases of the trajectory.

### First consultation

In the first consultation, in some cases, caregivers detected warning signs from the birth of their children/offspring. However, it took them weeks to several years to access specialists, particularly in the public sector, where they must be referred from primary to secondary or tertiary care. According to some interviewees, the delays were sometimes related to the fact that symptoms began mildly, so it took time for patients or caregivers to see the need to consult. In contrast, some participants reported that when the disease began in a severe manner with symptoms that caused widespread discomfort and even required hospitalisation in some cases, the consultation was much quicker, occurring within a few weeks or a few months from the first manifestation. According to the interviewees, there are differences between the experiences of participants affiliated with the public insurer or the private system in accessing care since, in the private system, individuals can directly access specialists, whereas in the public system, one requires referral from primary care and struggles with long waiting lists to reach an appointment with medical specialists:

“[…] well, he had facial paralysis at ten months old, they saw him at the primary health care centre and they told me that it was due to some strong wind, what do I know… After two and a half years or so, a doctor saw him and took me more seriously, as you can say, and she referred me to another doctor and there she said, “No, he needs a specialist, a neurologist” (Caregiver of patient without a diagnosis).

Patients and caregivers reported this first consultation as a slow and cumbersome process since health professionals—mainly those in primary care in the public system—are considered to lack training to identify symptoms of rare diseases. For this reason, patients perceived that their symptoms were ignored and, as a result, they do not manage to enter the health system, or they are referred to doctors who do not correspond to the specialist they require given the nature of their illness, which leads to a prolonged odyssey within the health system. According to the experience reported by several participants, this odyssey entails months or even years of navigating through the health system before being able to access a specialist who can give concrete answers to a patient’s discomfort:

I went to many doctors with my daughter, neurologists, and specialists, and no one had an answer. They just told me, “I’m going to keep looking.” No one told me, “Look, take her to that place where they can give you an answer” (Caregiver of a patient with a diagnosis).

Due to these challenges, participants reported that many patients covered by the public sector choose to seek care in the private sector. However, they must pay out-of-pocket for those consultations or sign up for private complementary insurance in addition to their public insurance:

“There was never anything in the public service, the waits were always long, it was almost impossible, or they didn’t know about the different pathologies, so we decided with my mother to start to look in the private sector, and there we made more progress” (Patient with a diagnosis).

### In search of a diagnosis

Once the first consultation takes place, patients are often asked to undergo a variety of laboratory or image testing in search of a diagnosis. From this stage onward, patients’ trajectories are very different depending on whether specific exams and diagnoses exist and whether there is a diagnosis and treatment for their conditions.

Reaching a diagnosis was associated with a series of factors, such as available technological advances and access to specialists who manage these diagnoses, including place of residence. The diagnostic tests that specialists requested varied from genetic tests to biopsies and imaging studies. There were several perceived barriers to exams among participants related to the healthcare system and the individual. Regarding the system, waiting lists varied from several months to years, either for subspecialists or for diagnosis, depending mainly on structural barriers such as access to genetic specialists, of which there are very few in Chile, access to tests, which are not available in every region, or the capacity to pay out-of-pocket for private care to accelerate the journey. Barriers pertaining to individuals and their families include a lack of information about these types of conditions, not having a relative with similar symptoms, residing in a geographical area that has lower availability of having these types of testing available, and perceiving symptoms as mild or vague to trigger a consultation. When symptoms are severe and require hospitalisation, the person is often tested quickly, and received a diagnosis sooner:

“We got here, I don’t know how I drove, I left my son without saying goodbye because I couldn’t take it anymore from the pain, I couldn’t even wear underwear anymore because everything was completely torn up to my groin, without skin. And my daughter took me to the clinic. I was admitted to the emergency room, and they admit me immediately. The doctors did not understand what it was; they called the head of dermatology, and he indicated to hospitalise me. Finally, they told me, “Look, there are many things here that are super confusing and that could be signs of many rare diseases, but the one that is at the base and that is your diagnosis is dermatomyositis” (Patient with a diagnosis).

### Trajectories of patients with a diagnosis and prescribed treatment

Among those who received a diagnosis and were prescribed treatment, the relevance of early detection of the disease was critical so that the disease was addressed before it could cause a significant deterioration of the body, allowing patients’ general state of health to improve:

“If this had not been detected early, I think I would have had permanent health problems; what I was telling you about the bones, I met girls who looked pregnant because they had an inflamed spleen and liver… they almost exploded, eh, wow, some girls were covered in bruises, who seemed a little malnourished, um, and that kind of thing” (Patient with a diagnosis).

According to the reports of participants with this type of trajectory, reaching a diagnosis opened a series of uncertain future paths for patients and their families. Once a diagnosis is reached, it is possible to access treatments and make personal therapeutic decisions in a more informed manner, in addition to making it possible for medical teams to offer treatments if they exist. This impacts the incorporation of changes in lifestyles necessary to cope with health conditions and thus increases the quality of life of the affected person.

“I feel that having a diagnosis not only influenced the fact that I could understand more about my illness, but it also changed my predisposition. In other words, understanding what one has is the first clue to finding more appropriate rhythms of life” (Patient with a diagnosis).

Some of those with a diagnosis requiring specific pharmacological treatments reported initial adjustment periods to the treatment to achieve the appropriate medication. Before following a specified treatment, other alternative therapies can also be attempted until the best therapeutic solution is achieved for that person. Additionally, only those with a condition covered by the public health system do not have to pay for it out-of-pocket. However, today, this system does not cover most conditions, which results in financial strain and emotional struggle for the patient and their family.

Based on participants’ perceptions and experiences, follow-ups with individuals who had a diagnosis and treatment for their rare condition depended on the degree of alleviation of symptoms that the treatment provided. If the treatment was effective and symptoms were mild, then the person continued the trajectory with that treatment. If the treatment did not alleviate symptoms or produced adverse side effects, then the trajectories became more complex, with additional consultations and frequent interactions with the healthcare system.

### Trajectories of patients with a diagnosis and no specific treatment

According to some interviewees, sometimes, receiving a precise diagnosis for an RD does not necessarily imply the availability of an effective treatment. Additionally, in many cases, these patients may have multiple diagnoses or more than one condition in the differential diagnosis, necessitating unique and complex models of treatments tailored for them. While some of these conditions require treatment, such treatments may not yet exist, resulting in the continuation of uncomfortable symptoms. This is perceived to affect patients’ well-being and quality of life and imposes an emotional burden on their caregivers.

In these cases, the search for treatment became an odyssey with periods of trial and error that could extend practically indefinitely. This may be experienced more frequently in those with multiple diagnoses than in those with a single diagnosis. For these patients, their continuous search process is interspersed with periods where possible diagnoses and new treatments are constantly reinvestigated depending on the level of expertise and treatments available in the healthcare system.

“About a year ago, I was diagnosed with dysautonomia; four years ago, I was diagnosed with Raynaud’s syndrome, but I’m still under investigation because I have a lot of pain and it’s not closely associated with pathologies. They have done many tests on me; they have told me that it could have been many things. Once they told me that it could be epilepsy, then they told me “No, it’s not epilepsy”; they did that to me to see if it was arthritis, if it was osteoarthritis, and there was none of those” (Patient with a diagnosis).

### Trajectories of patients without a diagnosis and treatment

When specific diagnostic tests are unavailable or do not provide conclusive information, health teams attempt a clinical diagnostic definition:

“The doctor told me, “You moved on to another group of patients who are patients with a rare disease without a diagnosis,” and that is my diagnosis. So, clinically, I have vascular Ehler-Danlos syndrome, and I believe that this is undeniable with all my clinical history, but genetically, I’m a part of the group of rare diseases without a diagnosis” (Patient without a confirmed diagnosis).

Regarding the specific impact of the diagnosis on individuals’ health, the idea is emphasised that reaching a diagnosis for a disease without treatment loses importance because it will not directly impact the final state of health of the patients, which shows the relevance of advancing the availability of medicines in cases where this corresponds:

“The diagnosis can be one thing, and the treatment, sometimes there is and other times there is not, so without treatment, I know it will not improve” (Caregiver of a patient without a confirmed diagnosis).

For people who do not yet have a diagnosis of a rare or uncommon disease, they still undergo treatments to alleviate symptoms as the search process for an aetiology continues. Treatments for symptoms vary from patient to patient. Nevertheless, in general, these approaches address many symptoms separately, which leads to the use of multiple medications, which come with the responsibility of following several treatments in parallel to improve health status.

There were two possible paths for those without a diagnosis. Some continue the search process to find answers for their health condition. Other patients and caregivers decide to stop the search and treatment process if they did not perceive noticeable improvements in either their quality of life or general state of health:

“I was looking for a solution for her [her daughter] for 16 years; the paediatrician told me to give myself a break because I had not reached any goal for 16 years. He told me, “You are tired; [daughter´s name] is also very tired”, and so far, no, there has been no diagnosis for her for 16 years, and unfortunately, I got sick there, and she [her daughter] had to be left aside” (Caregiver of patient without a confirmed diagnosis).

### Additional unmet needs in the three identified trajectories

This study offers some additional unique findings regarding the need for access to mental healthcare for all patients. This need was considered a priority in the three groups, given the impact that RDs have on the lives of patients and their families. In the public health system in particular, access to mental health for patients with rare diseases is restricted since it is not necessarily considered within the scope of treatment. Therefore, patients must receive a referral to access mental health services, a process that can take months to complete and, even access is granted, the duration of mental health services provision is brief:

“It’s quite complex to provide support because the mental health area does not have many resources, nor are there many people working there, so to get an appointment takes quite a while. Sometimes they do not accept patients referred from the hospital, so they have to go to primary care, get an appointment in primary care for mental health and mental health they have to see if they accept it or not” (Health professional).

These insufficient referral pathways mean that, in many cases, the responsibility for addressing the mental health of patients and caregivers falls on the treating doctors, who have little time in their consultation hours and do not have the necessary skills to address these needs. Patients and caregivers in the private system often must resort to paying out-of-pocket expenses to access mental health services, which poses a significant economic burden for their families. All of these findings indicate that mental health in rare diseases is largely unaddressed by both public and private health systems in Chile.

## Discussion

This study aimed to explore the therapeutic trajectories of RD in Chile by describing the experiences and perceptions of patients, caregivers, and medical teams regarding the initial symptoms, first consultation, testing, diagnosis, treatment, and follow-up. Our study revealed that the therapeutic trajectories of patients with rare, infrequent, or orphan diseases are similar at the initial symptom, first consultation, and diagnosis suspicion stages. However, their paths separate from each other from the exam stage onwards, with diverse experiences related to these journeys, largely based on whether they have a diagnosis and treatment available. This novel information helps understand how patients with unique health conditions, who often live outside the radar in healthcare decision-making for coverage and burden of disease efforts, navigate the Chilean healthcare system.

These findings are consistent with studies on therapeutic trajectories in RDs from other countries, where accessing diagnoses requires experiencing multiple interactions with the health system and different health providers [17, 18]. As in other studies, the initial symptoms, which are usually non-specific, are addressed through standard approaches to common diagnoses [26]. This is consistent with findings from this study, especially regarding the initial attention-seeking processes.

In search of a diagnosis, patients and their families make an average of at least six visits to different specialists [27]. According to previous studies, this implies extensive search periods, which have been reported up to five years or more [28]. The literature also indicates that caregivers perceive a particular value in reaching a diagnosis since, from their perspective, this allows them to understand the patient’s health situation and establish routes to follow in terms of care [29]. This international evidence aligns with the results of this study, where those who receive a diagnosis perceive that having one affords them some sense of certainty, especially when there is an accessible treatment available through the health system for their condition. The path of those with undiagnosed diseases is consistent with what has been found in other studies, where these trajectories are often reported as a “roller coaster” of discarded diagnostic suspicions, unexplained concerning symptoms, and incorrect diagnoses [29].

This exploratory qualitative study has several strengths. This is the first study in Chile to describe the unique therapeutic trajectories of patients and families living with RDs within the Chilean public and private healthcare systems. It included various perspectives from patients, caregivers, and healthcare workers. We used a flexible interview guide adapted for every case to uncover the perceptions and experiences of these patients and their families while navigating the Chilean healthcare system. We describe several general patterns and three unique trajectories based on the availability of diagnosis and treatment. We included patients and families living in different regions of the country with RD.

We also recognise certain limitations of this study in that we did not investigate specific rare conditions in detail, as this was the first study of its kind in Chile. This study did not delve in journeys pursued outside of the healthcare system, which is also relevant to patients and families seeking diagnosis and treatment. Due to the novelty of this kind of research in the country, the recruitment process was based on theoretical and practical criteria, which may inadvertently exclude patients and caregivers whose experience did not align with the study’s criteria. For instance, patients and caregivers who are not involved in the exome sequencing study or who were not related previously to other studies or with patients or caregivers’ organisations were not able to be invited, and their experiences might be underrepresented in the results section. This could imply potential biases by considering only patients and families that seek answers through innovative studies and group with others in organisations to find support. This may leave other patients’ and caregivers’ profiles with different therapeutic trajectories unconsidered. Also, for healthcare teams and professionals referred by patients and caregivers but also the ones contacted through convenience approach, the participants might be profiles more committed to accompanying the patient’s journey and people with better interpersonal relationships, which may influence the therapeutic trajectories considered in this research. This might leave other healthcare teams’ perspectives out of the picture. Additionally, it is relevant to point out that the characteristics of the Chilean health system may have shaped and influenced the trajectories found through the interviews. With 68% of the patients and caregivers participants of the research relying on public health coverage, the barriers and characteristics described in each phase of the trajectory might reflect the features of the public health system, like delays in care and the absence of required specialists. Also, the lack of public policy or specific legislation for addressing RDs impacts the therapeutic journeys of these patients, as a small number of them had the medication and treatments with coverage. Due to this situation, if the country would introduce new legislation on the matter, the therapeutic journeys identified through this research might vary.

It is important to emphasize that the qualitative paradigm does not aim to elucidate causal relationships; hence, findings from this study can be used to generate novel questions and hypotheses for future exploration that can also address some of its limitations, particularly concerning differences in the trajectories of patients and families with RD based on structural determinants such as their type of health insurance, region of residence, previous information about the condition, and availability for testing, diagnosis, and treatment.

This study sheds new light on the current knowledge available in the country about patients’ and families’ trajectories living with an RD in navigating the healthcare system in Chile. This is crucial given that RDs are often invisible in the health system since they affect a low percentage of the population. Revealing the experiences of patients and their families in the Chilean health system is a first step to move towards raising awareness about the need to incorporate RDs within different areas, such as in training health professionals or establishing clear referral pathways, to shorten delays in the identification of symptoms. For patients with a diagnosis and no treatment, the results highlight the need to generate support and accompaniment strategies for these cases, given the odyssey involved in searching for therapeutic alternatives to address the diagnosis. Finally, for the trajectories of patients without a diagnosis and treatment, the study accounts for the importance of patients and their families accessing support networks and advocates that coping strategies for their illnesses be covered by the health system.

In addition, the results highlight the importance of incorporating health services that consider the needs of patients and family members with RDs, especially in the field of mental health. The study results indicate that there is still a long way to go in clinical practice and health policies in this area, but highlighting the reality of rare diseases from the perspective of their protagonists is a necessary step for future advancements.

## Conclusions

In this qualitative study, health system performance is measured by its ability to leave no one behind, including people and families living with an RD who often do not have a precise diagnosis or treatment. Even when they do receive a diagnosis and treatment, these treatments are usually uncovered by health insurance due to prevalence-focused arguments and must be paid out-of-pocket by families. Rare conditions in Chile need further and urgent attention, including listening to the voices and experiences of those who live with them and their families.

## Electronic supplementary material

Below is the link to the electronic supplementary material.

Supplementary Material 1

Supplementary Material 2

## Data Availability

The datasets generated and analysed during the current study are not publicly available because they contain participants’ personal information. Nevertheless, they may be available from the corresponding author upon reasonable request.

## Data availability

The dataset supporting the conclusions of this article is available in https://drive.google.com/drive/folders/1H51KTsW3sSoadIbJBlsPONK2CPUhkeVf?usp=sharing.

## Abbreviations

FONASA: Fondo Nacional de Salud (National Health Fund)
ISAPRES: Pension Health Institutions
