# Supplementary material for: Therapeutic trajectories of families with rare diseases in Chile from the perspectives of patients, carers, and healthcare workers: a qualitative study
Source: Orphanet J Rare Dis. 2025 Feb 25;20:86. doi: 10.1186/s13023-025-03595-6 (PMC11863834; doi:10.1186/s13023-025-03595-6)
Supplement: Supplementary file 2 — Supplementary Material 2 [file 13023_2025_3595_MOESM2_ESM.docx]

November 9^th^, 2024

Dear Editorial Board of Orphanet Journal of Rare Diseases:

We are re-submitting our research manuscript “***Therapeutic trajectories of families with rare diseases in Chile from the perspectives of patients, carers, and healthcare workers: a qualitative study”*** to be considered for publication in Orphanet Journal of Rare Diseases.

We would like to express our gratitude to the reviewer for their insightful comments and suggestions. We have incorporated all her/his suggestions, which are noticeable in the document with track changes and also in this letter. We are optimistic that this revised version meets the necessary quality standards for acceptance.

Thank you for your consideration.

Báltica Cabieses, PhD

Alexandra Obach, PhD

Antonia Roberts,

Gabriela Repetto, PhD

| **Reviewer 2** | |
| --- | --- |
| I must say that the article is written remarkably and the English language used is also good but there are some issues that I would like you to acknowledge: | *We greatly appreciate the dedication put into your review. Your comments and suggestions have been immensely helpful in improving the manuscript.*  *All new information based on comments is noticeable in the document with track changes and in each response content in this letter.* |
| The paper acknowledges the lack of investigation into journeys pursued outside the healthcare system but does not delve deeply into potential biases. Reflecting on possible biases due to participant self-selection, particularly in qualitative research, would be useful. | *Thank you very much for the comment. To address this comment, we included an in-depth reflection of the potential biases that could emerge due to the methodological approach used.*  *The changes made can be read in the following paragraph (line 487): “Due to the novelty of this kind of research in the country, the recruitment process was based on theoretical and practical criteria, which may inadvertently exclude patients and caregivers whose experience did not align with the study's criteria. For instance, patients and caregivers who are not involved in the exome sequencing study or who were not related previously to other studies or with patients or caregivers’ organisations were not able to be invited, and their experiences might be underrepresented in the results section. This could imply potential biases by considering only patients and families that seek answers through innovative studies and group with others in organisations to find support. This may leave other patients' and caregivers’ profiles with different therapeutic trajectories unconsidered. Also, for healthcare teams and professionals referred by patients and caregivers but also the ones contacted through convenience approach, the participants might be profiles more committed to accompanying the patient's journey and people with better interpersonal relationships, which may influence the therapeutic trajectories considered in this research. This might leave other healthcare teams' perspectives out of the picture”* |
| More discussion of how the regional and socioeconomic factors within Chile's healthcare system may have influenced participant experiences would enhance the limitations and the expansion of the limitations section would be appreciated. | Thank you very much for the comment. To address this comment, we included a paragraph in the discussion section (line 501), reflecting on how the Chilean healthcare system may have influenced participant experiences.  *“Additionally, it is relevant to point out that the characteristics of the Chilean health system may have shaped and influenced the trajectories found through the interviews. With 68% of the patients and caregivers participants of the research relying on public health coverage, the barriers and characteristics described in each phase of the trajectory might reflect the features of the public health system, like delays in care and the absence of required specialists. Also, the lack of public policy or specific legislation for addressing RDs impacts the therapeutic journeys of these patients, as a small number of them had the medication and treatments with coverage. Due to this situation, if the country would introduce new legislation on the matter, the therapeutic journeys identified through this research might vary”* |
| Secondly, the study should incorporate visuals i.e. The therapeutic trajectories could be illustrated through flowcharts or diagrams to make the concept clearer to readers, particularly those unfamiliar with qualitative healthcare studies. | *Thank you very much for the comment. We find very important to illustrate the therapeutic journeys through visuals to complement the results section.*  *The flowchart that illustrates the therapeutic trajectories is located at the bottom of the article, after the references, with its figure title and legend section.* |
| Rest I would say that the study is well-designed and addresses an important gap in the literature, but with some structural and analytical improvements, it could make an even stronger contribution to the field of rare disease healthcare. | *Thank you very much for the comment. We appreciate the time you took to review our work. Your comments have helped to improve our work and deepen our reflections on its results. We hope this revised version meets the standards for acceptance.* |
